# Galloyl*-*Hexahydroxydiphenoyl (HHDP)-Glucose Isolated From *Punica granatum L*. Leaves Protects Against Lipopolysaccharide (LPS)-Induced Acute Lung Injury in BALB/c Mice

**DOI:** 10.3389/fimmu.2019.01978

**Published:** 2019-08-20

**Authors:** Aruanã Joaquim Matheus Costa Rodrigues Pinheiro, Aleff Ricardo Santos Mendes, Milena Dara Farias de Jesus Neves, Carla Máximo Prado, Márcia Isabel Bittencourt-Mernak, Fernanda Paula Roncon Santana, João Henrique G. Lago, Joicy Cortez de Sá, Claudia Quintino da Rocha, Eduardo Martins de Sousa, Valéria Costa Fontes, Marco Augusto Gregolin Grisoto, Angela Falcai, Lidio Gonçalves Lima-Neto

**Affiliations:** ^1^Programa de Pós-Graduação, Universidade CEUMA, São Luís, Brazil; ^2^Programa de Pós-Graduação da Rede BIONORTE, Universidade Estadual do Maranhão, São Luís, Brazil; ^3^Department of Biosciences, Federal University of São Paulo, Santos, Brazil; ^4^Department of Medicine, School of Medicine, University of São Paulo, São Paulo, Brazil; ^5^Centro de Ciências Naturais e Humanas, Universidade Federal do ABC, Santo André, Brazil; ^6^Departamento do Curso de Medicina, Universidade CEUMA, São Luís, Brazil; ^7^Departamento de Química, Universidade Federal do Maranhão, São Luís, Brazil; ^8^Programa de Pós-graduação, Mestrado em Meio Ambiente, Universidade CEUMA, São Luís, Brazil

**Keywords:** pomegranate, galloyl-HHDP-glucose, acute lung injury, anti-inflammatory effects, cytokines, leukocytes

## Abstract

The hydroalcoholic extract and ethyl acetate fraction of *Punica granatum* leaves have been known to exhibit anti-inflammatory activities. In this study, we investigated the therapeutic effects of galloyl-hexahydroxydiphenoyl (HHDP)-glucose isolated from pomegranate leaves on lipopolysaccharide (LPS)-induced acute lung injury (ALI) in mice. Male BALB/c mice were treated with different doses of galloyl-HHDP-glucose (5, 50, and 100 mg/Kg) or dexamethasone at 5 mg/Kg (*per os*) 6 h after intra-tracheal instillation of LPS. Vehicle-treated mice were used as controls. Twenty-four hours after LPS challenge, bronchoalveolar lavage fluid (BALF), and lung samples were collected for analyses. They were evaluated by monitoring the expression of NF-κB, JNK, and cytokine genes and proteins, as well as cell migration and lung function. All doses of galloyl-HHDP-glucose inhibited LPS-induced JNK and NF-κB activation. Likewise, the galloyl-HHDP-glucose-treated animals presented reduced expression of the TNF-α, IL-6, and IL-1β genes in the lungs and reduced TNF-α, IL-6, IL-1β, and IL-8 protein levels when compared with the vehicle-treated LPS-challenged mice. In addition, the ALI mice treated with galloyl-HHDP-glucose also presented reduced lung inflammatory cell accumulation, especially that of neutrophils, in their BALF and lungs. In addition, galloyl-HHDP-glucose treatment markedly ameliorated the LPS-induced pulmonary mechanism complications and attenuated weight loss. Overall, we showed for the first time that galloyl-HHDP-glucose protects against ALI, and may be useful for treating ALI and other inflammatory disorders.

## Introduction

Acute respiratory distress syndrome (ARDS) is a life-threatening respiratory failure and one of the most challenging clinical conditions. It results in nearly 75,000 deaths annually, and is associated with high morbidity and mortality rates (1). There are more than 3 million ARDS cases annually worldwide, and they account for 10% of intensive care unit admissions (1).

Acute respiratory distress syndrome is characterized by an acute diffused and inflammation lung injury, leading to intense trans-epithelial leukocyte infiltration, exudate accumulation in the lungs, loss of integrity of the alveolar-capillary membrane, and tissue damage (2). Acute lung injury (ALI) induced by lipopolysaccharides (LPS) in mice is a well-accepted model for investigating ARDS because it mimics the inflammatory and histological changes observed in this disease. In this model, LPS stimulates alveolar macrophages via the TLR4/CD14/MD2 complex and the MyD88-dependent response, and activates nuclear factor kappa B (NF-κB) and c-Jun N-terminal kinase (JNK) that regulate the secretion of inflammatory mediators such as cytokines (e.g., IL-8, TNF-α, IL-1β, and IL-6) (3). The secreted inflammatory mediators then trigger neutrophil infiltration into alveolar space (4). This intense inflammatory response is associated with ARDS exacerbation (5). Despite the advances in ARDS diagnosis and treatment in the last 5 years, treatment options are limited and no pharmacology treatments aimed at the underlying pathology have proven effective.

*Punica granatum L*. (pomegranate) has been shown to possess wound-healing, antimicrobial, antioxidant, and anti-inflammatory properties (6–8). The anti-inflammatory properties of pomegranate have been exploited in traditional medicine for the treatment of inflammatory disorders. We previously demonstrated that the hydroalcoholic extract of pomegranate leaves inhibits TNF-α production and decreases neutrophil migration in a rat model of LPS-induced acute peritonitis (8). Additionally, the ethyl acetate fraction of the hydroalcoholic extract of pomegranate leaves was effective in treating LPS-induced ALI in mice (9). Some pharmacologically useful compounds, especially gallotannins, have been identified in the ethyl acetate fraction of pomegranate leaves (9). Galloyl-hexahydroxydiphenoyl (HHDP)-glucose identified in the ethyl acetate fraction is a hydrolysable tannin, shown to also be present in pomegranate juice, emblic leafflower fruits, and *Eucalyptus globulus* Labill bark (10, 11). However, its pharmacological activities remain undetermined.

The effects of galloyl-HHDP-glucose on the inflammatory response, especially in acute lung inflammation, have not been reported. Hence, in this study, we assessed the anti-inflammatory potential of galloyl-HHDP-glucose isolated from pomegranate leaves, in an LPS-induced ALI mouse model.

## Materials and Methods

### Isolation and Identification of Galloyl-HHDP-Glucose

**Figure 1:**
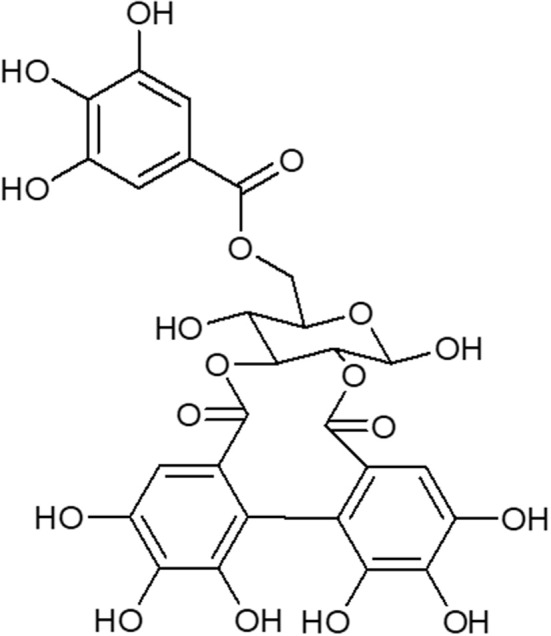
Structure of galloyl-HHDP-glucose isolated from the hydroalcoholic extract of *Punica granatum L*.

The hydroalcoholic extract and ethyl acetate fraction of pomegranate leaves were obtained as previously described (8). All compounds in the ethyl acetate fraction were identified as previously described (9). The ethyl acetate fraction was separated by chromatography on a silica gel (230–400 mesh) column (8 × 100 cm), and eluted with crescent polarity mixtures of n-hexane/ethyl-acetate and ethyl-acetate/methanol. The fractions obtained were subjected to another round of chromatography resulting in 6 fractions, with one of them containing galloyl-HHDP-glucose of >95% purity. The structure was determined using HPLC-DAD-ESI-IT/MS analysis as previously described (9). The data obtained were compared to those verified in a previous study which investigated the chemical structure of galloyl-HHDP-glucose (Figure 1).

### Ethics Statement

All animal care and experimental procedures were conducted in compliance with the rules of the British Pharmacological Society's Ethics Committee. All experimental protocols were approved by the internal ethical committee of the University of São Paulo (#962/18).

### Animals

The animals were housed under controlled light (12 h light/12 h dark; lights on at 8 am) and temperature conditions (23 ± 1°C), with free access to water and food. All animals were obtained from the Animal Facility of the University of São Paulo, São Paulo, Brazil.

### Induction of LPS-Induced ALI and Pharmacological Treatments

Sixty-six non-fasted male inbred BALB/c mice (6–8 weeks) were randomly distributed into six groups (*n* = 10/group) for lung and bronchoalveolar lavage fluid (BALF) analyzes. The mice were anesthetized with isoflurano (2%) and ALI was induced by intratracheal instillation of LPS (*E. coli* 026: B6L3755, 20 μL of 5 mg/mL in 0.9% NaCl per animal equivalent to 5 mg/Kg). Vehicle-treated mice were used as controls (20 μL of 0.9% NaCl per animal). Mice received a single oral administration (p.o.) of vehicle (saline, 1 mL/kg), Galloyl-HHDP-glucose (5–100 mg/kg at 1 mL/kg) or dexamethasone (5 mg/kg at 1 mL/kg) 6 h after LPS administration.

### Respiratory Mechanics and Body Weight (BW) Evaluation

Twenty-four hours after LPS challenge, mice were weighed, anesthetized with thiopental (80 mg/kg), tracheostomized, and connected to a ventilator (FlexiVent, SCIRED, Montreal, Canada) maintained at a tidal volume and respiratory frequency of 10 mL/kg and 150 breathes/min, respectively, for respiratory mechanics evaluation. The respiratory system elastance (Ers) and resistance (Rrs) were obtained using the equation of motion of the respiratory system as previously described (4), and the tissue damping (Gtis) and elastance (Htis) parameters were obtained from Zrs data by applying the constant phase model as previously described (4). Body weight (BW) was recorded at 0 h, and at 24 h post LPS challenge.

### Total and Differential Leukocyte Counting in BALF Samples

The BALF in anesthetized animals was collected as previously described (9). An aliquot (100 μL) of the BALF was used for quantification of leukocytes, and the rest was centrifuged at 400 × g for 10 min at 4°C. The supernatant was immediately frozen and stored at −80°C for further analysis. In a separate series of experiments, the lungs were collected from animals whose BALF was not collected, and processed for histological analysis. Total cell counts were determined in a Neubauer chamber, using a microscope (Zeiss Axio Imager Z2 upright microscope; Carl Zeiss, Göttingen, Germany), after diluting an aliquot of the BALF with Türk solution (1:20). Another aliquot (50 μL) was used for differential cell counts as previously described (9).

### RT-qPCR Assays With Lung Tissues

The left lung (~100 mg) was mechanically homogenized with a using a TissueLyser II bead mill (Qiagen, Valencia, CA), with 5-mm stainless steel beads. Total RNA was extracted using an RNAeasy Mini Kit (Qiagen, Hilden, Germany). Samples were treated with DNase (Qiagen, Valencia, CA) and then, the quantity of purified RNA was measured at 260/280 absorbance using a NanoDrop Lite Spectrophotometer (ThermoFisher Scientific, USA). The RNA (~200 ng) was reverse-transcripted using the High Capacity cDNA Transcription Kit (ThermoFisher Scientific, USA) to produce complementary DNA (cDNA). TNF-α, IL-6, IL1-β, and IL-10 mRNA expression was determined by RT-qPCR as described previously (9). In brief, the qPCR assays were performed in a 25 μL reaction mixture containing primers, 1x GoTaq® qPCR Master Mix (Promega Corporation), and 4 μL of cDNA. The qPCR assays were carried out in the QuantStudio™ 6 Flex (Thermo Fisher Scientific, USA) using the following program: one cycle at 95°C for 10 min, followed by 40 cycles at 95°C for 10 s and 60°C for 1 min. GAPDH mRNA was used as an endogenous reference gene and the mRNA relative expression was calculated using the 2^−Δ*Ct*^ method. Results represent the mean values obtained from two independent experiments, with assays performed in triplicate.

### Lung Histology

The right lung lobes were fixed in 4% formaldehyde for 24 h and then was embedded in paraffin. After deparaffinization and dehydration as previously described (12), sections (4 μm) were stained with hematoxylin and eosin and analyzed by microscopy under 100x, and 400x magnification using an Axio Imager Z2 Zeiss microscope (Carl Zeiss). The criteria used in the histological evaluation were (1) alveolar and peribronchiolar inflammatory infiltrate, (2) alveolar septum thickening, and (3) edema. The following inflammatory infiltrate scores were established: absence (not observed in any field), mild (1–3 observed fields), moderate (4–6 observed fields), and intense (over 7 observed fields). The density of leukocytes/unit area (130 μm^2^) was determined by counting the number of cells on the integrating eyepiece that fell into areas of the bronquioli and distal lung parenchyma using the Image J Program. All measurements were performed in 10 random sections of 130 μm^2^ per animal at 400x magnification for leukocyte count; and the researcher who performed the analysis was unaware of the experimental group designation. Two independent researchers performed blinded analyses; if there was discordance, a third researcher performed the analysis.

### Measurement of Cytokine Levels in the Lung Tissue

The left lung (~100 mg) was homogenized with a Polytron PTA 20S (Brinkmann Instruments, model PT 10/35) in 1 ml of protein extraction buffer before centrifugation at 12,000 g/4°C for 15 min. Total protein content in the lung homogenate was determined using Bradford reagent (Protein Assay, Bio-Rad), and the resulting values were used to normalize the values of the specific proteins detected by ELISA in the lung tissue. The lung TNF-α, IL-6, IL1-β, IL-8, and IL-10 protein levels were analyzed using ELISA kits (R&D Systems, Minneapolis, USA), in accordance with the manufacturer's instructions. Sample readings for each cytokine were compared with a standard curve (0–800 pg/mL, serial two-fold dilution). Results are expressed as protein levels, in pg/mL. All experiments were performed in triplicates.

### Measurement of Total Protein Levels in Lung Homogenates

Total protein levels were measured using the Bradford method, using the standard Quick Start Bradford Protein Assay Kit (Bio-Rad, Hercules, CA, USA), in accordance with the manufacturer‘s instructions. Absorbance was measured in an automated spectrophotometer at 595 nm. Readings obtained were compared with those of a standard curve of albumin (0–0.8 mg/mL). All experiments were performed in triplicates.

### Western Blot Analysis in Lung Homogenate

NF-κB subunit p65 and β-actin expression in lung tissue homogenates were quantified in lung homogenates that were treated with Laemmli sample buffer containing dithiothreitol (Bio-Rad). Protein concentrations of the supernatants were determined using the Bradford assay, and an equal amount of total protein from each sample (50 g) was treated with Laemmli buffer containing dithiothreitol (100 mM). The samples were then subjected to SDS-PAGE (10% bis-acrylamide). Protein electrotransfer of proteins from the gel to a nitrocellulose membrane (Bio-Rad) was performed for 90 min at 15 V (constant). Non-specific protein binding to nitrocellulose was reduced by preincubating the membrane overnight at 4°C in blocking buffer (5% milk/Tris-buffered saline with Tween 20). The antibodies used for the immunoblot were anti-p65-NF-κB and anti-phosphorylate p65-NF-κB (both 1:1,000) (Cell Signaling, Danvers, MA) and anti-β-actin (1:1,000) (Sigma Aldrich, St. Louis, MO) diluted in blocking buffer overnight at 4°C. The membranes were then washed for 30 min with Tris-buffered saline with Tween 20. The bound antibodies were detected with horseradish peroxidase-conjugated anti-IgG (1:10,000) and visualized by chemiluminescence using UVItec (UVItec, Cambridge, MA). The band intensities were quantified using the UVItec Image Program. Phosphorylated NF-κB expression was normalized to total NF-κB expression.

### RAW 264.7 Macrophage Culture

RAW 264.7 cells were cultured and maintained in DMEM medium (Thermo Fisher Scientific, USA) containing 10% fetal bovine serum and 1% antibiotic solution: 1,000 U/mL penicillin G and 100 U/mL streptomycin sulfate (Thermo Fisher Scientific, USA).

### Cell Viability

Cells (5 × 10^5^ cells/well) were treated with EAFPg (125, 250, 500, and 1,000 μg/mL). Then, cells were incubated for 48 h at 37°C and 5% CO_2_. Vehicle (DMSO 1% in PBS)-treated cells were used as controls. Each culture well was delicately washed once with phosphate buffered saline solution [PBS: NaCl 0.8%, KCl 0.02%, KH_2_PO_4_ 0.02%, Na_2_HPO_4_ 0.12%, pH 7.3] and the cell viability was determined by MTT assay. In brief, cells were incubated with 100 μL of MTT at a final concentration of 5 mg/mL (Sigma Aldrich, USA) for 3 h and then centrifuged at 1,800 rpm for 10 min at room temperature to remove the supernatant. Afterwards, the formed formazan crystals were dissolved in 1 mL of dimethyl sulfoxide (DMSO) for 15 min and absorbance read was determined by 570 nm with 655 nm as the wavelength reference. The results were expressed as a percentage of the maximal value of the positive control and reported as means of three independent assays and the standard deviation.

### Statistical Analysis

The normality of variables was evaluated using the Kolmogorov–Smirnov normality test. The statistical differences between the groups were analyzed using One-way ANOVA followed by Tukey test (for the comparison of three or more groups for Gaussian data) or Kruskal-Wallis followed by Dunn method (for non-parametric data). Percentages of inhibition were calculated as the mean of the inhibitions obtained for each individual experiment. *p* values < 0.05 were considered statistically significant. Data are expressed as mean ± standard deviation (SD).

## Results

### Galloyl-HHDP-Glucose Treatment Reduced Phosphorylation of the NF-κB p-65 Subunit and JNK in the Isolated Lung Tissues

**Figure 2:**
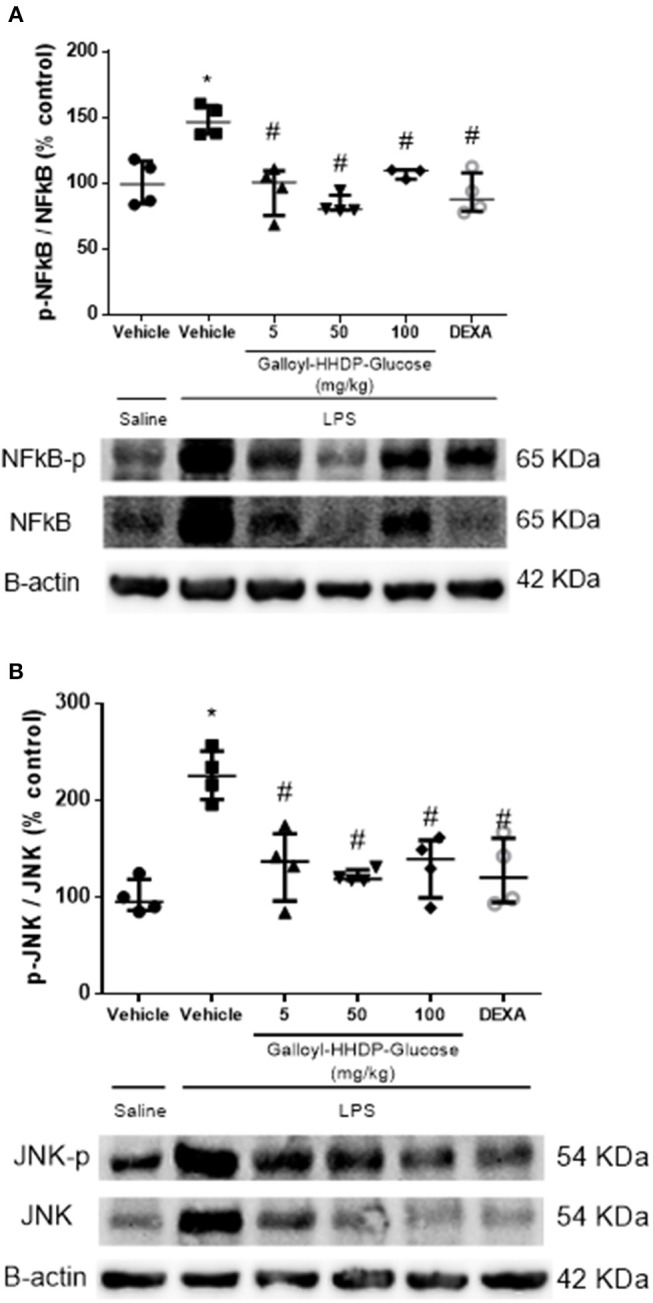
Effects of galloyl-HHDP-glucose treatments on the activation of NF-κB **(A)** and JNK **(B)** in the lungs of BALB/c mice. BALB/c mice were randomly allocated to 6 groups (*n* = 4/group). Mice received galloyl-HHDP-glucose (5, 50, and 100 mg/kg; p.o), dexamethasone (DEXA; 5 mg/kg; p.o), or vehicle (PBS, 10 mL/kg; p.o). The results were calculated as % increase relative to that in the control. β-actin was used as a constitutive protein. The values are presented as median and interquartile. Significances were calculated by Kruskal–Wallis followed by Dunn multiple comparison test analysis of the groups vs. saline-injected non-treated mice (^*^p < 0.05) and vs. lipopolysaccharide (LPS)-installed non-treated mice (^#^*p* < 0.05). The gel is representative of results that were obtained in an experiment repeated four times.

We evaluated the total and phosphorylated levels of the NF-κB p-65 subunit and JNK in lung homogenates, to analyze the possible mechanisms involved in the anti-inflammatory effects of galloyl-HHDP-glucose (Figure 2). Animals with ALI exhibited an increased expression of phosphorylated p-65-NF-κB/total p-65-NF-κB, compared with the saline-treated group. In contrast, all doses of galloyl-HHDP-glucose blocked LPS-induced activation of p-65-NF-κB. The galloyl-HHDP-glucose-treated animals also presented significantly reduced lung JNK expression that was initially induced by LPS, compared with the vehicle-treated mice.

### Galloyl-HHDP-Glucose Reduced TNF-α, IL-1β, and IL-6 mRNA Expression in the Isolated Lung Tissues

**Figure 3:**
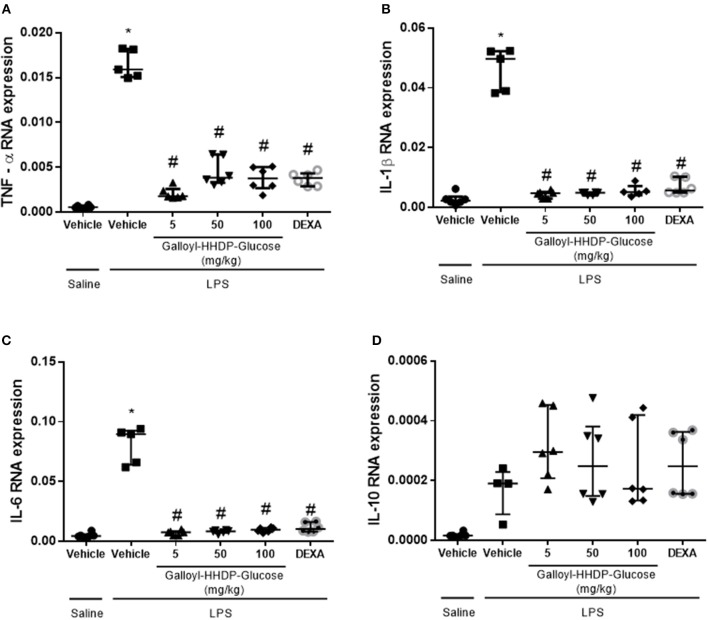
Effects of galloyl-HHDP-glucose treatment on TNF-α **(A)**, IL-1β **(B)**, IL-6 **(C)**, and IL-10 **(D)** gene expression in isolated lung tissues of BALB/c mice. BALB/c mice were randomly allocated to 6 groups (*n* = 6/group). Mice received galloyl-HHDP-glucose (5, 50, and 100 mg/kg; p.o), dexamethasone (DEXA; 5 mg/kg; p.o), or vehicle (PBS; p.o). The values are presented as median and interquartile range. Significances were calculated by Kruskal–Wallis followed by Dunn multiple comparison test analysis of the groups vs. saline-injected non-treated mice (^*^p < 0.05) and vs. lipopolysaccharide (LPS)-installed non-treated mice (^#^*p* < 0.05).

LPS instillation significantly increased TNF-α, IL-1β, and IL-6 mRNA expression in the lungs (Figures 3A–C). All test concentrations of galloyl-HHDP-glucose or 5 mg/kg dexamethasone significantly down-regulated lung TNFα, IL-1β, and IL-6 gene expression in mice with ALI. The administered doses of galloyl-HHDP-glucose and dexamethasone had no effects on IL-10 gene expression (Figure 3D).

### Galloyl-HHDP-Glucose Treatment Reduced Pro-inflammatory Protein Levels in the Isolated Lung Tissues

**Figure 4:**
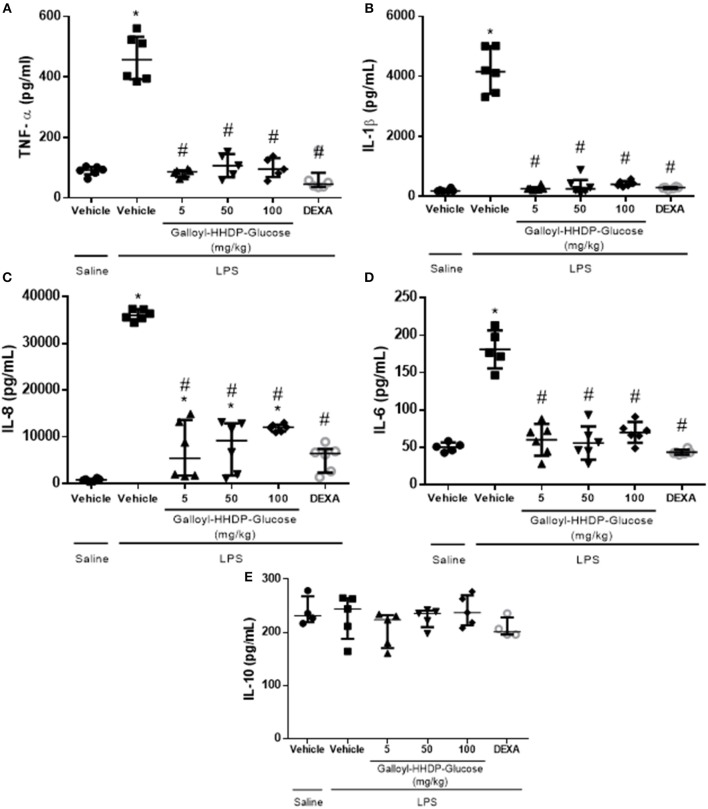
Effects of galloyl-HHDP-glucose treatments on TNF-α **(A)**, IL-1β **(B)**, IL-8 **(C)**, IL-6 **(D)**, and IL-10 **(E)** levels in homogenized isolated lung tissues of BALB/c mice. BALB/c mice were randomly allocated to 6 groups (*n* = 6/group). Mice received galloyl-HHDP-glucose (5, 50, and 100 mg/kg; p.o), dexamethasone (DEXA; 5 mg/kg; p.o), or vehicle (PBS; p.o). The values are presented as median and interquartile. Significances were calculated by Kruskal–Wallis followed by Dunn multiple comparison test analysis of the groups vs. saline-injected non-treated mice (^*^p < 0.05) and vs. lipopolysaccharide (LPS)-installed non-treated mice (^#^*p* < 0.05).

LPS-induced increases in IL-1β, TNF-α, IL-6, and IL-8 protein levels in the lung homogenate samples were down-regulated by treatment with either galloyl-HHDP-glucose or dexamethasone (Figures 4A–D). None of the doses of galloyl-HHDP-glucose or dexamethasone had any effect on IL-10 levels (Figure 4E).

### Galloyl-HHDP-Glucose Reduced Body Weight Loss and LPS-Induced Pulmonary Leukocyte Migration

**Figure 5:**
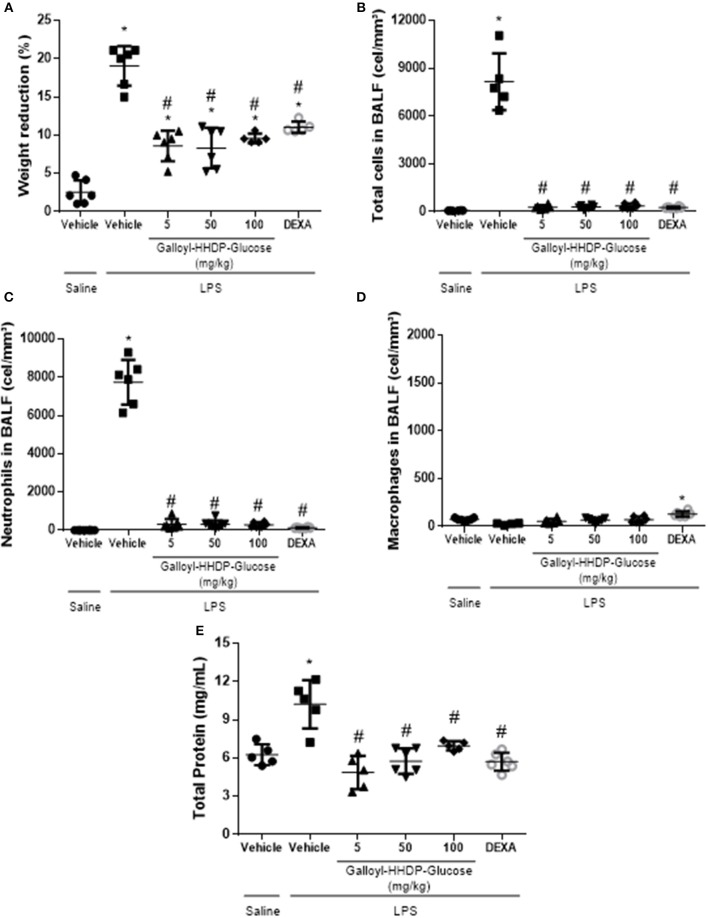
Analyses of body weights of mice. **(A)** Effects of galloyl-HHDP-glucose treatment on the total number of leukocytes **(B)**, neutrophils **(C)**, or macrophages **(D)** in bronchoalveolar lavage fluid (BALF), and total proteins in lung **(E)**. BALB/c mice were randomly allocated to 6 groups (*n* = 6/group). Mice received galloyl-HHDP-glucose (5, 50, and 100 mg/kg; p.o), dexamethasone (DEXA; 5 mg/kg; p.o), or vehicle (PBS; p.o). The values are presented as mean and SD. Significances were calculated by One-way ANOVA followed by Tukey test analysis of the groups vs. saline-injected non-treated mice (^*^p < 0.05) and vs. lipopolysaccharide (LPS)-installed non-treated mice (^#^*p* < 0.05).

Figure 5 shows the effects of galloyl-HHDP-glucose (5, 50, and 100 mg/kg) and dexamethasone (5 mg/kg) on body weight loss and the inflammatory cells in the BALF of mice with LPS-induced ALI. Galloyl-HHDP-glucose-treated animals presented significantly reduced body weight loss at all doses tested, compared with those of the vehicle-treated ALI mice (Figure 5A). Pulmonary inflammation was also evaluated in the BALF (Figures 5B–D). Mice with LPS-induced ALI had significantly increased total leukocytes and neutrophils in their BALF, compared with those in the non-ALI group (Figures 5B,C). In contrast, treatment with all doses of galloyl-HHDP-glucose significantly diminished total inflammatory cell and neutrophil numbers in the BALF, compared with those of the vehicle-treated animals (Figures 5B,C). A similar effect was noted for LPS-challenged mice treated with dexamethasone. No differences were observed in the number of macrophages between groups.

### Total Protein Level Decreased in the Lungs of LPS-Treated Mice

Total protein levels were augmented in lung samples obtained from mice with ALI (Figure 5E). On the other hand, treatments with galloyl-HHDP-glucose significantly decreased total protein levels in LPS-treated mice, compared with those in vehicle-treated LPS-challenged mice (*p* < 0.05).

### Galloyl-HHDP-Glucose Prevented Leukocyte Migration to the Lungs of Mice With ALI

**Figure 6:**
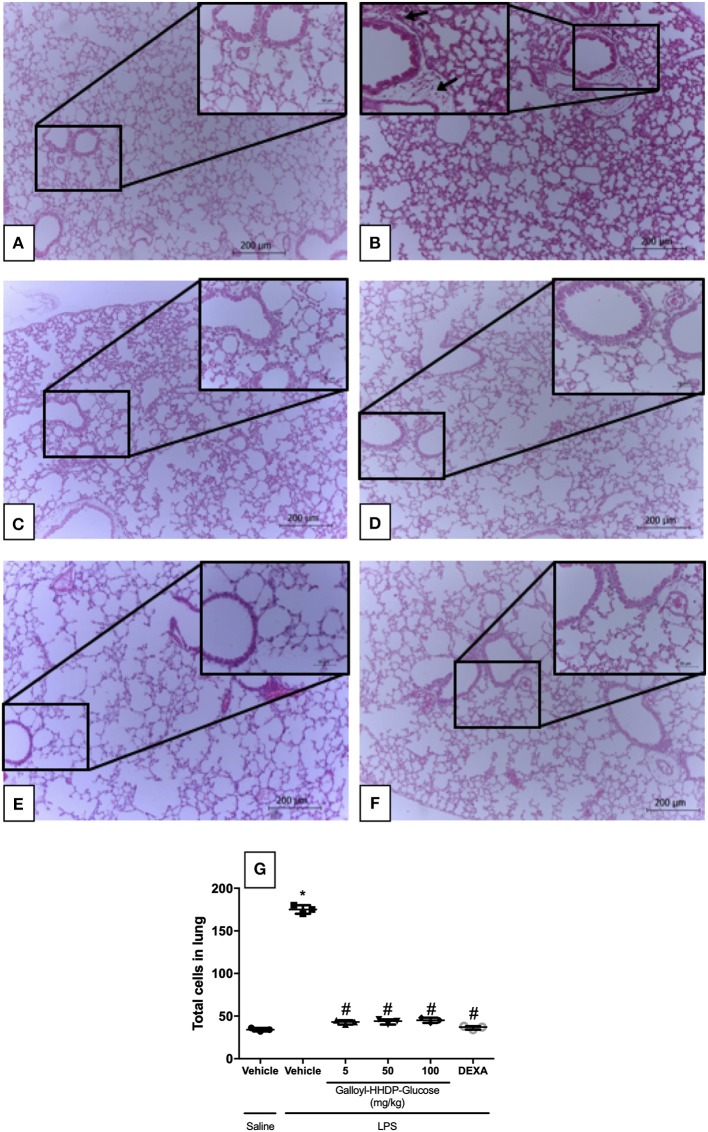
Effect of galloyl-HHDP-glucose treatment on leukocyte accumulation in the lungs of mice treated with saline-injected non-treated mice **(A)**, lipopolysaccharide (LPS)-installed non-treated mice **(B)**, 5 mg/kg galloyl-HHDP-glucose and LPS **(C)**, 50 mg/kg galloyl-HHDP-glucose and LPS **(D)**, 100 mg/kg galloyl-HHDP-glucose and LPS **(E)**, 5 mg/kg dexamethasone and LPS **(F)**, and total cells in lung **(G)**. Black arrows: bronchiolar inflammatory cell infiltrates. Sections (4 μm) were stained with hematoxylin and eosin, and viewed with a microscope at 100x and 400x magnification respectively. Significances were calculated by One-way ANOVA followed by Tukey test analysis of the groups vs. saline-injected non-treated mice (^*^p < 0.05) and vs. lipopolysaccharide (LPS)-installed non-treated mice (^#^*p* < 0.05).

Animals with ALI exhibited an intense inflammatory-cell influx surrounding the bronchi (Figure 6B). Galloyl-HHDP-glucose treatment (5, 50, and 100 mg/kg) significantly reduced leukocyte infiltration, compared with infiltration in vehicle-treated LPS mice (Figures 6C–E,G). Dexamethasone-treated animals also displayed attenuated lung inflammation (Figure 6F).

### Galloyl-HHDP-Glucose Ameliorates LPS-Induced Pulmonary Mechanic Complications

**Figure 7:**
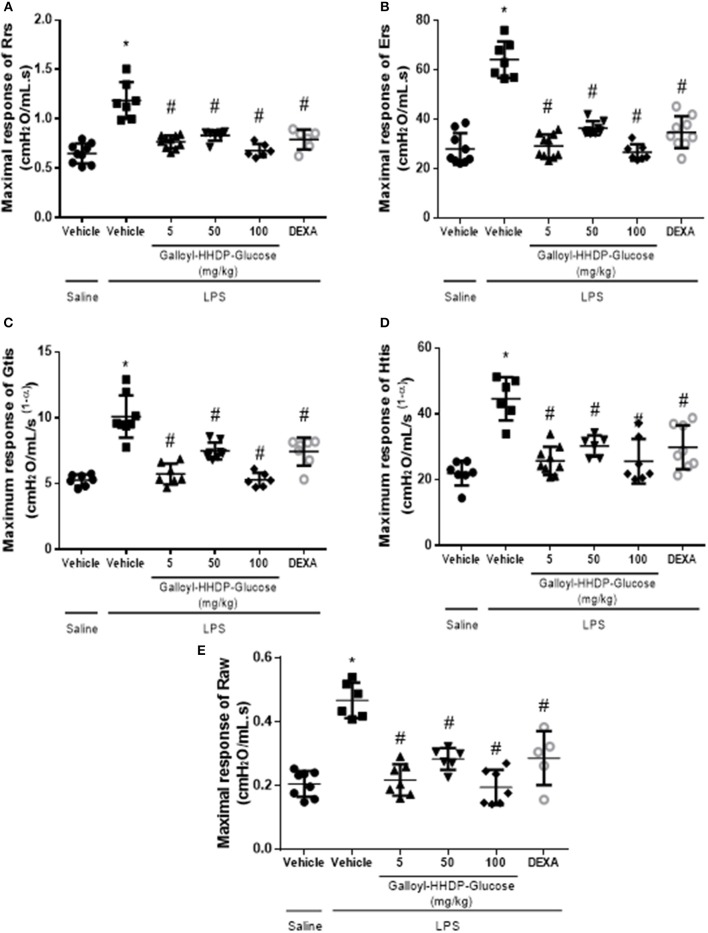
Efficacy of galloyl-HHDP-glucose treatments in reducing respiratory system and lung tissue elastance in lipopolysaccharide (LPS)-induced ALI mice. The respiratory system resistance (Rrs) and elastance (Ers), tissue damping (Gtis) and elastance (Htis), and airway resistance (Raw) **(A–E)**. The mice received different doses of galloyl-HHDP-glucose (5, 50, and 100 mg/kg) 6 h after intra-tracheal instillation of LPS. The values are presented as mean and SD. Significances were calculated by One-way ANOVA followed by Tukey test. Significances were calculated by One-way ANOVA followed by Tukey test analysis of the groups vs. saline-injected non-treated mice (^*^p < 0.05) and vs. lipopolysaccharide (LPS)-installed non-treated mice (^#^*p* < 0.05).

The pulmonary mechanic data are shown in Figure 7. The increased Rrs, Ers, Gtis, Htis, and airway resistance (Raw) parameters in mice with ALI reflect LPS-induced pulmonary mechanic alterations. On the other hand, galloyl-HHDP-glucose treatments significantly reduced all parameters in LPS-challenged animals, compared with those in the vehicle-treated LPS-challenged mice (*p* < 0.05, Figures 7A–E).

### Effect of Galloyl-HHDP-Glucose on Cell Viability of RAW 264.7 Cells

**Figure 8:**
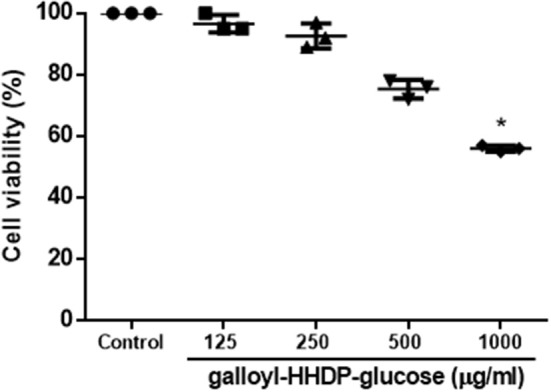
Effect of Galloyl-HHDP-glucose for cell viability of RAW 264.7 cells. Results are expressed as means followed by standard deviation of three independent experiments performed in triplicate. (^*^) indicates statistically significant different compared to control group by Kruskal–Wallis followed by Dunn multiple comparison test analysis.

Cell viability was not affect by Galloyl-HHDP-glucose, except for the higher tested concentration (1,000 μg/mL), as shown in Figure 8.

## Discussion

Extracts prepared from pomegranate peel, seeds, and leaves have been shown to possess anti-inflammatory properties (6, 12–14). We demonstrated previously that the hydroalcoholic extract of pomegranate leaves attenuates LPS-induced peritonitis by decreasing the accumulation of leukocytes and release of pro-inflammatory mediators in the peritoneum (8). We also showed that its ethyl acetate fraction protects mice from ALI by reducing lung inflammation. However, the pharmacological properties of galloyl-HHDP-glucose, a molecule present in pomegranate leaves has not been documented. This study therefore investigated the effects of galloyl-HHDP-glucose on mice with LPS-induced ALI.

In animal models, LPS-induced ALI mimics the inflammatory and histological changes observed in ARDS (15, 16). LPS stimulates acute inflammation through the activation of lung-resident macrophages, which in turn activate intracellular mediators (17). The transcription factor NF-κB regulates innate and adaptive immune functions, and serves as a pivotal mediator of inflammatory responses (3). We observed that galloyl-HHDP-glucose blocked the activation of NF-κB, thus, regulating lung inflammation. LPS-activated macrophages induces a phosphorylation of NF-κB developing a proinflammatory profile by gene expressing and releasing cytokines as observed in animals with ALI (18). IKB (inhibitor of Kinase B) is a key factor in NF-κB activation (19). Moreover, the mitogen activated protein kinase (MAPK) is one of the IKK activators by different stimulis such as TLRs, IL-1β, TNFα, and others (19). Three main MAPKinase pathways have been described in mammalian cells: the extracellular signal-regulated kinases (ERKs), the JNK pathway and the p38 MAPK pathway (20). JNK is activated in lung tissue of ARDS mice and its inhibition by specific inhibitors may significantly improve the pulmonary histopathology and lung permeability in rats (21). Reinforcing the importance of JNK in the modulation of inflammation in ALI, we showed that LPS activates JNK in lung and Galloyl-HHDP-glucose treatment is able to inhibits lung JNK phosphorylation similar to dexamethasone.

In animals with ALI, LPS-activated macrophages induce the phosphorylation of NF-κB, which leads to development of a proinflammatory profile via expression of cytokine genes and release of cytokines (18). Therefore, to further evaluate the effects of galloyl-HHDP-glucose treatment in lung inflammation, we measured the inflammatory markers TNF-α, IL1-β, IL-8, and IL-6. The LPS-induced increase in the gene expression of proinflammatory cytokines was significantly reduced by galloyl-HHDP-glucose treatment. In a previous study, the hydroalcoholic extract of pomegranate leaves reduced TNF-α mRNA expression in peritoneal leukocytes obtained from rats with LPS-induced peritonitis (8). Galloyl-HHDP-glucose may be one of the compounds related to the anti-inflammatory effects previously observed in treatment with the hydroalcoholic extract and ethyl acetate fraction of pomegranate.

TNF-α induces the expression of adhesion molecules on endothelial cells. IL-8 is the primary cytokine involved in the recruitment of neutrophils mainly via the MAPK pathway. In ALI, neutrophils are the first cells to be recruited to the lungs (22). In this study, we demonstrated that galloyl-HHDP-glucose reduces neutrophil migration into the alveoli. Marques et al. (8) showed that the number of peritoneal leukocytes, especially neutrophils, is reduced in hydroalcoholic extract-treated rats with acute peritonitis (8).

In patients with ARDS, the intensity of signs and symptoms depends on the cause and severity of the disease. However, variations in intensity are mainly associated with lung dysfunction caused by an intense inflammatory response (23). Hence, inflammation-modulating molecules are widely employed in the treatment of ARDS patients (1). In this study, we observed that galloyl-HHDP-glucose suppresses lung inflammation in the airways of mice intratracheally challenged with LPS. Moreover, this treatment attenuated the loss of weight induced by LPS suggesting that this treatment not only improve lung function but also reduced the systemic effects of LPS instillation. We showed the main therapeutic effect exhibited by Galloyl-HHDP-glucose was to reduce the cytokine production by suppressing NF-κB and MAPKinase activation; these results strongly suggest that Galloyl-HHDP-glucose possesses potent anti-inflammatory actions in the lungs. Thus, we suggest that Galloyl-HHDP-glucose impairs the generation of these inflammatory mediators by interfering with these signaling pathways in leukocytes.

The pulmonary dysfunction caused by the intense inflammatory response is related to lung failure, which results in death of ARDS patients. Elevations in the parameters used as indicators for LPS-induced pulmonary mechanic complications were attenuated by galloyl-HHDP-glucose, improving lung function in this animal model.

## Conclusion

Galloyl-HHDP-glucose exerts anti-inflammatory effects that improve lung function in an LPS-induced ALI mouse model. Therefore, it could possibly be used to treat ALI and even other inflammatory disorders.

## Data Availability

All datasets generated for this study are included in the manuscript.

## Ethics Statement

All animal care and experimental procedures were conducted in compliance with the rules of the British Pharmacological Society's Ethics Committee. All experimental protocols were approved by the internal ethical committee of the University of São Paulo (#962/18).

## Author Contributions

AP, CP, EdS, and LL-N conceived and designed the work. AP, AM, MN, MB-M, FS, JL, JdS, CdR, VF, and LL-N contributed to data acquisition and performed the experiments. EdS, AF, and LL-N drafted the manuscript. AP, JL, CdR, and MB-M isolated galloyl-HHDP-glucose. AP, CP, MG, AF, JdS, and LL-N critically revised the manuscript for important intellectual content and gave final approval for the version to be published.

### Conflict of Interest Statement

The authors declare that the research was conducted in the absence of any commercial or financial relationships that could be construed as a potential conflict of interest.
