# Corrigendum: Galloyl*-*Hexahydroxydiphenoyl (HHDP)-Glucose Isolated From *Punica granatum L*. Leaves Protects Against Lipopolysaccharide (LPS)-Induced Acute Lung Injury in BALB/c Mice

**DOI:** 10.3389/fimmu.2019.02727

**Published:** 2019-11-27

**Authors:** Aruanã Joaquim Matheus Costa Rodrigues Pinheiro, Aleff Ricardo Santos Mendes, Milena Dara Farias de Jesus Neves, Carla Máximo Prado, Márcia Isabel Bittencourt-Mernak, Fernanda Paula Roncon Santana, João Henrique G. Lago, Joicy Cortez de Sá, Claudia Quintino da Rocha, Eduardo Martins de Sousa, Valéria Costa Fontes, Marco Augusto Gregolin Grisoto, Angela Falcai, Lidio Gonçalves Lima-Neto

**Affiliations:** ^1^Programa de Pós-Graduação, Universidade CEUMA, São Luís, Brazil; ^2^Programa de Pós-Graduação da Rede BIONORTE, Universidade Estadual do Maranhão, São Luís, Brazil; ^3^Department of Biosciences, Federal University of São Paulo, Santos, Brazil; ^4^Department of Medicine, School of Medicine, University of São Paulo, São Paulo, Brazil; ^5^Centro de Ciências Naturais e Humanas, Universidade Federal do ABC, Santo André, Brazil; ^6^Departamento do Curso de Medicina, Universidade CEUMA, São Luís, Brazil; ^7^Departamento de Química, Universidade Federal do Maranhão, São Luís, Brazil; ^8^Programa de Pós-graduação, Mestrado em Meio Ambiente, Universidade CEUMA, São Luís, Brazil

**Keywords:** pomegranate, galloyl-HHDP-glucose, acute lung injury, anti-inflammatory effects, cytokines, leukocytes

In the published article, there was an error in affiliation “3.” The affiliation “Departamento do Curso de Farmácia, Faculdade Pitágoras, São Luis, Brazil,” should be removed. All remaining affilations have thus been renumbered accordingly.

The authors apologize for this error and state that this does not change the scientific conclusions of the article in any way. The original article has been updated.

